# Supplementary material for: Quality of reported ages: A robust re-modification in Total Modified Whipple’s Index
Source: PLoS One. 2025 Aug 22;20(8):e0330519. doi: 10.1371/journal.pone.0330519 (PMC12373282; doi:10.1371/journal.pone.0330519)
Supplement: S1 Appendix — (DOCX) [file pone.0330519.s001.docx]

Appendix

Table A1: Digit-specific modified Whipple’s indexes (Wi) and total modified Whipple’s indexes (Wtot) for India, Turkey and Pakistan.

| Country and year(s) | Digit-specific Modified Whipple index  Wi | | | | | | | | | | |  | Total modified Whipple index |
| --- | --- | --- | --- | --- | --- | --- | --- | --- | --- | --- | --- | --- | --- |
|  | W0 | W1 | W2 | W3 | W4 | W5 | W6 | W7 | W8 | W9 | ΣWi | | Wtot |
| India | | | | | | | | | | | | |  |
| 2005–2006 | 2.069 | 0.499 | 1.091 | 0.811 | 0.688 | 2.031 | 0.807 | 0.658 | 1.017 | 0.524 | 10.194 | | 4.210 |
| 2015–2016 | 1.643 | 0.673 | 1.165 | 0.921 | 0.820 | 1.671 | 0.845 | 0.777 | 1.061 | 0.686 | 10.262 | | 2.817 |
| 2019–2021 | 1.491 | 0.690 | 1.190 | 0.959 | 0.871 | 1.516 | 0.881 | 0.815 | 1.088 | 0.771 | 10.272 | | 2.300 |
| Turkey | | | | | | | | | | | | |  |
| 1993 | 1.529 | 0.753 | 0.955 | 1.312 | 0.910 | 1.265 | 0.858 | 0.959 | 1.028 | 0.741 | 10.309 | | 1.959 |
| 1998 | 1.306 | 0.795 | 1.042 | 1.209 | 1.028 | 1.142 | 0.950 | 0.919 | 1.079 | 0.830 | 10.299 | | 1.311 |
| 2003 | 1.172 | 0.862 | 1.035 | 1.242 | 1.048 | 1.118 | 0.887 | 0.944 | 1.049 | 0.952 | 10.310 | | 1.019 |
| 2008 | 1.168 | 0.823 | 1.072 | 1.161 | 1.052 | 1.135 | 0.920 | 0.932 | 1.096 | 0.913 | 10.272 | | 1.098 |
| 2013 | 1.166 | 0.965 | 1.031 | 1.206 | 0.993 | 1.053 | 0.938 | 0.998 | 1.023 | 0.900 | 10.273 | | 0.685 |
| 2018 | 1.251 | 0.811 | 1.150 | 1.124 | 1.017 | 1.130 | 0.938 | 0.958 | 1.092 | 0.827 | 10.297 | | 1.230 |
| Pakistan | | | | | | | | | | | | |  |
| 1990–1991 | 2.677 | 0.405 | 0.837 | 0.725 | 0.604 | 2.439 | 0.724 | 0.548 | 0.817 | 0.356 | 10.132 | | 6.100 |
| 2006–2007 | 2.048 | 0.542 | 0.973 | 0.867 | 0.776 | 1.909 | 0.831 | 0.654 | 1.038 | 0.579 | 10.216 | | 3.774 |
| 2012–2013 | 1.847 | 0.481 | 1.174 | 0.900 | 0.794 | 1.796 | 0.832 | 0.726 | 1.076 | 0.615 | 10.242 | | 3.544 |
| 2017–2018 | 1.504 | 0.668 | 1.183 | 1.008 | 0.860 | 1.545 | 0.870 | 0.813 | 1.127 | 0.711 | 10.289 | | 2.446 |
| Data Source: Standard DHS datasets for India (2005–2021), Turkey (1993–2018), and Pakistan(1990–91) [18] | | | | | | | | | | | | |  |

Table A2: Digit-specific Original Whipple’s indexes (WIi) and Robust modified Whipple’s indexes (RMWI) for India, Turkey, and Pakistan.

| Country and year(s) | Digit-specific Whipple index  WIi | | | | | | | | | |  | Robust modified Whipple index |
| --- | --- | --- | --- | --- | --- | --- | --- | --- | --- | --- | --- | --- |
|  | WI0 | WI1 | WI2 | WI3 | WI4 | WI5 | WI6 | WI7 | WI8 | WI9 | ΣWIi | RMWI |
| India | | | | | | | | | | | |  |
| 2005–2006 | 2.012 | 0.425 | 0.908 | 0.777 | 0.725 | 2.086 | 0.870 | 0.678 | 1.033 | 0.486 | 10.000 | 4.262 |
| 2015–2016 | 1.596 | 0.584 | 0.978 | 0.882 | 0.850 | 1.719 | 0.899 | 0.794 | 1.058 | 0.641 | 10.000 | 2.746 |
| 2019–2021 | 1.461 | 0.603 | 1.001 | 0.891 | 0.886 | 1.547 | 0.936 | 0.840 | 1.104 | 0.731 | 10.000 | 2.225 |
| Turkey | | | | | | | | | | | |  |
| 1993 | 1.407 | 0.664 | 0.813 | 1.270 | 0.937 | 1.365 | 0.883 | 0.942 | 1.018 | 0.701 | 10.000 | 2.122 |
| 1998 | 1.197 | 0.678 | 0.865 | 1.169 | 1.079 | 1.237 | 1.011 | 0.921 | 1.073 | 0.770 | 10.000 | 1.531 |
| 2003 | 1.079 | 0.754 | 0.864 | 1.236 | 1.096 | 1.207 | 0.919 | 0.939 | 1.016 | 0.891 | 10.000 | 1.268 |
| 2008 | 1.080 | 0.707 | 0.898 | 1.128 | 1.098 | 1.221 | 0.981 | 0.948 | 1.085 | 0.853 | 10.000 | 1.225 |
| 2013 | 1.132 | 0.902 | 0.925 | 1.162 | 0.989 | 1.084 | 0.937 | 0.976 | 1.010 | 0.883 | 10.000 | 0.777 |
| 2018 | 1.213 | 0.728 | 1.016 | 1.036 | 1.015 | 1.164 | 0.978 | 0.956 | 1.102 | 0.792 | 10.000 | 1.090 |
| Pakistan | | | | | | | | | | | |  |
| 1990–1991 | 2.559 | 0.350 | 0.720 | 0.707 | 0.643 | 2.546 | 0.773 | 0.550 | 0.822 | 0.328 | 10.000 | 6.215 |
| 2006–2007 | 1.973 | 0.450 | 0.773 | 0.815 | 0.807 | 1.978 | 0.905 | 0.678 | 1.075 | 0.546 | 10.000 | 4.054 |
| 2012–2013 | 1.736 | 0.392 | 0.922 | 0.866 | 0.853 | 1.904 | 0.920 | 0.759 | 1.090 | 0.558 | 10.000 | 3.460 |
| 2017–2018 | 1.401 | 0.548 | 0.935 | 0.973 | 0.913 | 1.651 | 0.956 | 0.850 | 1.123 | 0.650 | 10.000 | 2.349 |
| Data Source: Standard DHS datasets for India (2005–2021), Turkey (1993–2018), and Pakistan(1990–91) [18] | | | | | | | | | | | | |

Table A3: Digit-specific modified Whipple’s indexes (Wi), digit-specific Original Whipple’s indexes (WIi), total modified Whipple’s indexes (Wtot), and Robust modified Whipple’s indexes (RMWI) for simulated data Series.

| Data Series | Digit-specific Modified Whipple index  Wi | | | | | | | | | | |  | | Total modified Whipple index | |
| --- | --- | --- | --- | --- | --- | --- | --- | --- | --- | --- | --- | --- | --- | --- | --- |
|  | W0 | W1 | W2 | W3 | W4 | W5 | W6 | W7 | W8 | W9 | ΣWi | | Wtot | |  |
| S1 | 1.123 | 0.963 | 1.103 | 1.264 | 0.843 | 0.957 | 0.952 | 1.024 | 1.023 | 1.018 | 10.271 | | 0.839 | |  |
| S2 | 0.953 | 1.217 | 1.082 | 0.833 | 1.153 | 1.099 | 1.066 | 0.960 | 0.825 | 1.121 | 10.309 | | 1.166 | |  |
| S3 | 1.014 | 1.132 | 0.953 | 1.103 | 1.173 | 0.904 | 0.974 | 0.974 | 1.102 | 0.979 | 10.308 | | 0.740 | |  |
| S4 | 1.050 | 1.164 | 1.109 | 1.072 | 1.102 | 0.806 | 1.201 | 1.051 | 0.865 | 0.950 | 10.370 | | 1.129 | |  |
| S5 | 0.983 | 1.116 | 1.024 | 1.160 | 1.071 | 0.955 | 1.041 | 1.015 | 0.952 | 1.012 | 10.328 | | 0.548 | |  |
| S6 | 0.998 | 1.058 | 1.150 | 1.044 | 1.053 | 1.007 | 0.981 | 0.968 | 1.032 | 1.011 | 10.303 | | 0.408 | |  |
| S7 | 0.983 | 1.075 | 1.127 | 1.081 | 1.041 | 1.019 | 0.990 | 0.993 | 1.060 | 0.964 | 10.334 | | 0.473 | |  |
| S8 | 0.969 | 1.091 | 1.104 | 1.103 | 1.014 | 1.005 | 1.026 | 0.992 | 0.985 | 1.028 | 10.316 | | 0.426 | |  |
| S9 | 0.964 | 1.098 | 1.112 | 1.110 | 1.044 | 1.011 | 0.980 | 1.006 | 1.035 | 0.963 | 10.325 | | 0.510 | |  |
| S10 | 0.999 | 1.033 | 1.116 | 1.130 | 1.044 | 1.001 | 0.992 | 0.990 | 1.015 | 1.007 | 10.326 | | 0.365 | |  |
| Data Series | Digit-specific Whipple index  WIi | | | | | | | | | | |  | | Robust modified Whipple index | |
|  | WI0 | WI1 | WI2 | WI3 | WI4 | WI5 | WI6 | WI7 | WI8 | WI9 | ΣWIi | | RMWI | |  |
| S1 | 1.229 | 1.012 | 1.036 | 1.060 | 0.723 | 0.867 | 0.867 | 1.012 | 1.084 | 1.108 | 10.000 | | 1.084 | |  |
| S2 | 0.962 | 1.167 | 0.962 | 0.741 | 1.073 | 1.088 | 1.073 | 0.978 | 0.820 | 1.136 | 10.000 | | 1.073 | |  |
| S3 | 1.053 | 1.093 | 0.869 | 0.957 | 1.037 | 0.869 | 0.973 | 0.973 | 1.140 | 1.037 | 10.000 | | 0.718 | |  |
| S4 | 1.058 | 1.118 | 1.025 | 0.936 | 1.034 | 0.800 | 1.179 | 1.015 | 0.880 | 0.955 | 10.000 | | 0.859 | |  |
| S5 | 0.958 | 1.050 | 0.913 | 1.060 | 1.034 | 0.980 | 1.044 | 1.012 | 0.945 | 1.005 | 10.000 | | 0.410 | |  |
| S6 | 1.030 | 1.023 | 1.042 | 0.944 | 0.989 | 0.975 | 0.968 | 0.963 | 1.038 | 1.028 | 10.000 | | 0.323 | |  |
| S7 | 0.988 | 0.999 | 0.997 | 0.970 | 0.984 | 1.015 | 1.005 | 1.005 | 1.068 | 0.970 | 10.000 | | 0.185 | |  |
| S8 | 0.975 | 1.045 | 0.986 | 0.992 | 0.958 | 0.998 | 1.019 | 1.001 | 0.989 | 1.038 | 10.000 | | 0.204 | |  |
| S9 | 0.962 | 1.040 | 1.005 | 1.014 | 0.998 | 1.014 | 0.984 | 1.002 | 1.021 | 0.960 | 10.000 | | 0.192 | |  |
| S10 | 1.000 | 0.982 | 1.005 | 1.018 | 0.995 | 1.000 | 0.990 | 0.990 | 1.015 | 1.005 | 10.000 | | 0.086 | |  |

Table A4: Raw datasets of Turkey Demographic and Health Survey [1993–2018]

|  | | Survey years | | | | | |
| --- | --- | --- | --- | --- | --- | --- | --- |
| Current age | 1993 | | 1998 | 2003 | 2008 | 2013 | 2018 |
| 21 | 651 | | 659 | 915 | 740 | 763 | 566 |
| 22 | 799 | | 702 | 1014 | 828 | 750 | 581 |
| 23 | 796 | | 699 | 998 | 794 | 750 | 603 |
| 24 | 688 | | 719 | 895 | 762 | 677 | 591 |
| 25 | 773 | | 710 | 899 | 810 | 722 | 608 |
| 26 | 588 | | 679 | 838 | 749 | 673 | 523 |
| 27 | 661 | | 593 | 758 | 791 | 694 | 573 |
| 28 | 643 | | 602 | 730 | 763 | 691 | 558 |
| 29 | 489 | | 493 | 726 | 672 | 576 | 445 |
| 30 | 786 | | 642 | 875 | 740 | 735 | 646 |
| 31 | 449 | | 392 | 632 | 598 | 692 | 456 |
| 32 | 506 | | 521 | 698 | 641 | 714 | 563 |
| 33 | 614 | | 570 | 671 | 599 | 697 | 498 |
| 34 | 445 | | 512 | 613 | 635 | 660 | 516 |
| 35 | 613 | | 570 | 707 | 725 | 692 | 591 |
| 36 | 431 | | 470 | 487 | 554 | 649 | 525 |
| 37 | 501 | | 487 | 579 | 553 | 539 | 509 |
| 38 | 570 | | 561 | 679 | 640 | 639 | 610 |
| 39 | 392 | | 379 | 660 | 531 | 631 | 479 |
| 40 | 625 | | 547 | 701 | 660 | 682 | 666 |
| 41 | 287 | | 343 | 487 | 361 | 555 | 394 |
| 42 | 392 | | 466 | 560 | 582 | 609 | 500 |
| 43 | 487 | | 451 | 618 | 554 | 568 | 483 |
| 44 | 291 | | 371 | 536 | 578 | 481 | 486 |
| 45 | 472 | | 453 | 599 | 576 | 571 | 589 |
| 46 | 284 | | 336 | 431 | 474 | 436 | 471 |
| 47 | 283 | | 266 | 476 | 409 | 532 | 458 |
| 48 | 346 | | 406 | 515 | 568 | 575 | 517 |
| 49 | 198 | | 251 | 370 | 388 | 446 | 358 |
| 50 | 445 | | 394 | 455 | 561 | 606 | 613 |
| 51 | 289 | | 279 | 364 | 368 | 537 | 360 |
| 52 | 339 | | 284 | 443 | 486 | 509 | 555 |
| 53 | 369 | | 256 | 452 | 481 | 636 | 519 |
| 54 | 247 | | 221 | 384 | 389 | 437 | 468 |
| 55 | 578 | | 358 | 470 | 516 | 488 | 575 |
| 56 | 272 | | 223 | 280 | 335 | 380 | 468 |
| 57 | 235 | | 211 | 268 | 287 | 461 | 402 |
| 58 | 257 | | 244 | 328 | 364 | 399 | 552 |
| 59 | 172 | | 179 | 218 | 245 | 360 | 327 |
| 60 | 655 | | 439 | 360 | 364 | 558 | 539 |
| 61 | 159 | | 132 | 188 | 195 | 273 | 268 |
| 62 | 214 | | 190 | 213 | 224 | 277 | 446 |
| 63 | 239 | | 172 | 248 | 252 | 376 | 408 |
| 64 | 162 | | 161 | 223 | 196 | 279 | 320 |

List of Abbreviations

DHS Demographic and Health Survey

DI Digit index

P Probability

WI Whipple index

Wtot Total modified Whipple’s Index

RMWI Robust modified Whipple’s Index
