# Supplementary material for: Quality of reported ages: A robust re-modification in Total Modified Whipple’s Index
Source: PLoS One. 2025 Aug 22;20(8):e0330519. doi: 10.1371/journal.pone.0330519 (PMC12373282; doi:10.1371/journal.pone.0330519)
Supplement: Attachment — Submitted filename: Review Report PONE-D-25-11698 NSB.pdf [file pone.0330519.s003.pdf]

Review of Manuscript

Quality of reported ages: A robust re-modification in Total Modified Whipple's Index

Summary and Overall Impression

I would like to commend the authors for this well-researched and timely manuscript. The paper addresses a fundamental and persistent challenge in demographic data analysis the accurate measurement of age misreporting. The work makes a significant and valuable contribution by proposing a new, robust summary index (RW_{tot}) for measuring age heaping across all ten terminal digits.

The importance of this paper lies not only in the introduction of a new method but also in its rigorous and clear critique of the mathematical shortcomings in existing comprehensive indices. Specifically, the authors' deconstruction of the Total Modified Whipple Index (W_{tot}) , as proposed by Spoorenberg(2007) based on Noumbissi's work, is a critical insight for the field. This manuscript is a fine example of methodological refinement that promises to improve the quality and reliability of demographic analysis.

A major strength of the manuscript is its clear and logical deconstruction of the evolution of Whipple's Index, from its original form to its various modifications. The critique of Noumbissi's (1992) modification and the subsequent Spoorenberg index(W_{tot}) is particularly compelling. The authors effectively demonstrate the mathematical flaw wherein the formulas for terminal digits 1,2,3 and 4 lead to an overestimation of preference.

The authors employ a highly effective multi-pronged validation approach that substantially strengthens their claims.

Suggestions for Improvement

The manuscript is strong, and the following suggestions are offered in a constructive spirit to enhance its clarity, impact, and utility for the research community.

Refine the Terminology

While the name "remodified of total modified Whipple's Index" (and its abbreviation RW_{tot}) is accurate in describing its lineage, it is somewhat cumbersome and may hinder recall and adoption. To facilitate easier reference and citation, the authors might consider a more concise and memorable name. Suggestions include:

- Comprehensive Rogers-Whipple Index (CRWI), to credit the methodological foundation.
- Ten-Digit Whipple Index (TDWI) , to highlight its key feature.
- Robust General Whipple Index (RGWI) , to emphasize its stability.

A more distinct name could help establish the index as a new standard in literature.

Address the Linearity Assumption Trade-off

The paper correctly notes that Noumbissi's method was developed partly to address the "crude" 10-year linearity assumption of Roger's index , opting instead for a more granular 5-year assumption. The proposed RW_{tot}, by building on Roger's framework, reverts to this 10-year assumption. The manuscript would be strengthened by a brief discussion explicitly acknowledging this trade-off. The authors should clarify why accepting the broader 10-year linearity assumption is a worthwhile compromise to achieve a mathematically consistent and robust summary index that covers all ten digits without distortion.

Elaborate on the Proposed Quality Criteria

The paper proposes a new set of quality criteria for the RW_{tot} index in Table 2. This is an excellent and necessary step for practical application. However, the derivation of these specific thresholds (e.g. ,"Moderate" for a value of 1.00-1.99, corresponding to 5-9.99% of people reporting an incorrect age) is not detailed. The manuscript would be significantly enhanced by adding a paragraph explaining the rationale behind these ranges. How were these thresholds calibrated? A clearer justification would increase transparency and boost the confidence of researchers looking to apply these standards in their own work.

Enhance Data Visualization

The figures are informative, but the comparison between the competing indices could be made more direct and impactful. I would suggest creating a single, combined chart for a key dataset that plots the digit preference indices side-by-side. For example , using the Turkey 1993 data from Table 4 , a bar chart could directly contrast Noumbissi's W; with the proposed WI; for each digit. This would provide a powerful , at-a-glance visualization of the overestimation issue in the former method and the more plausible distribution of the latter.

Concluding Remarks and Recommendation

In conclusion , this manuscript presents a valuable, robust, and well-validated tool for demographic analysis. It successfully identifies and rectifies a significant mathematical flaw in a widely used method for assessing age data quality. The proposed RW_{tot} index is a clear improvement, offering researchers a more reliable and comprehensive measure of age misreporting.

The paper's strengths are substantial , and the identified areas for improvement are addressable through minor revisions that would further clarify its contributions and enhance its practical utility.

Recommendation: I recommend this manuscript for publication. The proposed index represents a significant and needed improvement over existing methods for assessing the overall quality of age reporting. I am confident that this work will be of great interest and utility to the demographic research community. Publication is recommended following minor revisions to address the constructive suggestions outlined in this review.
