# Supplementary material for: Quality of reported ages: A robust re-modification in Total Modified Whipple’s Index
Source: PLoS One. 2025 Aug 22;20(8):e0330519. doi: 10.1371/journal.pone.0330519 (PMC12373282; doi:10.1371/journal.pone.0330519)
Supplement: Attachment — Submitted filename: Plos One_Dr Farooq.docx [file pone.0330519.s002.docx]

To be accepted for publication in *PLOS One*, research articles must satisfy the following criteria:

1. **The study presents the results of original research.**

Yes. The manuscript presents an original methodological contribution to demographic measurement by proposing a new index, “RWtot”, for assessing the quality of age reporting. The innovation lies in its mathematically robust formulation and its ability to overcome limitations in existing indices like the Total Modified Whipple Index (Wtot) and Noumbissi’s digit-specific indices. The proposed method enhances accuracy and interpretability for assessing age heaping across all digits.

2. **Results reported have not been published elsewhere.**

The approach and formulation of the RWtot index appear to be novel and are applied to publicly available Demographic and Health Survey (DHS) datasets and simulated data for validation.

3. **Experiments, statistics, and other analyses are performed to a high technical standard and are described in sufficient detail.**

Yes. The authors provide clear mathematical derivations, define all index formulas, and apply the method to extensive real-world datasets from India, Pakistan, and Turkey. They also include simulation studies that support the index's robustness. The statistical procedures and underlying assumptions (e.g., equal terminal digit probabilities) are well justified.

4. **Conclusions are presented in an appropriate fashion and are supported by the data.**

Yes. The conclusions follow logically from the empirical and simulated results. The authors demonstrate that RWtot produces more balanced and accurate assessments of digit preferences. Figures and tables effectively support these claims.

5. **The article is presented in an intelligible fashion and is written in standard English.**

Generally yes. The article is intelligible and the structure is coherent. However, minor language improvements are recommended.

6. **The research meets all applicable standards for the ethics of experimentation and research integrity.**

Yes. The research uses publicly available DHS data. No human or animal subjects are directly involved.

7. **The article adheres to appropriate reporting guidelines and community standards for data availability.**

Yes. Data used in the study are available through the DHS Program website.

**Summary of the Article**

This paper introduces a more precise measure for detecting age heaping in demographic data by proposing a re-modified version of the Total Modified Whipple’s Index (RWtot). The authors critique the limitations of existing age-quality measures—particularly the Whipple Index and Spoorenberg's Total Modified Whipple Index—arguing that these either focus too narrowly on digits ending in 0 and 5 or suffer from biased digit-specific weighting. The proposed RWtot, grounded in the original Whipple’s structure, ensures equal treatment of all terminal digits (0–9) and addresses methodological inconsistencies in earlier adaptations. The method is tested using DHS data from India, Pakistan, and Turkey and validated with simulated datasets.

**General Assessment**

This is an excellent and well-structured contribution to the field of demographic data quality. The authors succeed in both identifying a nuanced problem—bias in existing digit-specific indices—and offering a mathematically sound and interpretable solution. The paper is methodologically rigorous, clearly written, and practically relevant for demographers and statistical agencies working with census or survey data. Its novelty lies in the construction of RWtot using consistent probabilistic and mathematical logic, while retaining computational simplicity.

**3. Major Comments**

**Main Strengths:**

The article makes a clear and justified methodological advancement by proposing RWtot. The RWtot is built on a solid critique of the denominator issues in Noumbissi's Wi index and addresses them using a uniform framework (equations 15–24). The authors have provided convincing empirical tests using large DHS datasets and simulation studies. The work is timely and relevant for low- and middle-income countries, where age misreporting is common and demographic indicators depend on clean data.

**Suggestions for Strengthening the Paper:**

Robustness Checks: Consider including additional countries (e.g., sub-Saharan Africa) to show geographic robustness.

Sensitivity to Age Range: While the focus is rightly on 23–62, a brief note on the applicability (or adjustment needs) for other age ranges could broaden utility.

Terminology Consistency: Throughout the article, ensure consistent use of symbols (e.g., WI, Wi, RWtot) to avoid reader confusion, especially in equations and figures.

**4. Minor Comments**

Ensure that all figures and tables are referenced in the text and are clearly labeled in the final version.

**5. Recommendation**

Accepted with Minor Revisions

The manuscript makes a meaningful and methodologically robust contribution to demographic measurement. The paper is accepted with minor revision, especially focus the stylistic and presentation will further improve the clarity and accessibility to the readers.
