# Supplementary material for: Quality of reported ages: A robust re-modification in Total Modified Whipple’s Index
Source: PLoS One. 2025 Aug 22;20(8):e0330519. doi: 10.1371/journal.pone.0330519 (PMC12373282; doi:10.1371/journal.pone.0330519)
Supplement: Attachment — Submitted filename: Response to Reviewers_11698.docx [file pone.0330519.s005.docx]

**Research Article**

**Quality of reported ages: A robust re-modification in Total Modified Whipple’s Index**

Dear Editor,

We are grateful for the opportunity to revise the manuscript titled **“Quality of reported ages: A robust re-modification in Total Modified Whipple’s Index” (PONE-D-25-11698)**. We thank the academic editor and the reviewers for their constructive and detailed comments on the original manuscript. We have carefully considered every suggestion and have revised the manuscript accordingly. Please find below our detailed response to the editor’s and reviewers’ comments.

**Note: Editor’s and reviewers’ comments are left-aligned and italicized; authors’ responses are justified and in normal text.**

**Response to Editor**

We thank the editor for his constructive and detailed comments on the original version of the manuscript. Below, we provide a point-by-point response to each comment.

*Please review your reference list to ensure that it is complete and correct. If you have cited papers that have been retracted, please include the rationale for doing so in the manuscript text, or remove these references and replace them with relevant current references. Any changes to the reference list should be mentioned in the rebuttal letter that accompanies your revised manuscript. If you need to cite a retracted article, indicate the article’s retracted status in the References list and also include a citation and full reference for the retraction notice.*

We thank the academic editor for this important observation. We carefully reviewed and updated the reference list to ensure that all entries are complete, accurate, and properly formatted. To the best of our knowledge, there is **NO** retracted article cited in text as well as in the reference list.

***# Additional Editor Comments:***

*Please incorporate more recent publications that are relevant to the theme of the study. The manuscript lacks a strong theoretical foundation in its current form.*

We sincerely thank the academic editor for this important feedback. We have addressed the comment in two ways:

Recent Literature: We have reviewed and incorporated several recent publications that are directly relevant to age misreporting and age data quality. These references have been added to the Introduction section (Paragraphs 1 and 2) to strengthen the manuscript’s connection with current research.

Theoretical Foundation: To enhance the theoretical basis of our study, we have added a paragraph on the theoretical foundation that clarifies how our proposed Robust General Whipple Index (RGWI) builds upon to address the limitations of earlier indices. This has been added in the revised version of the manuscript under the Introduction section, subsection: Theoretical Foundation.

**Response to Reviewer # 1**

We sincerely thank **Reviewer 1** for the positive and encouraging feedback on our manuscript. We also appreciate the thoughtful suggestions, which have helped us to improve the manuscript further. Below, we respond point by point to each suggestion.

*Suggestions for Strengthening the Paper:*

*Robustness Checks: Consider including additional countries (e.g., sub-Saharan Africa) to show geographic robustness.*

Thank you for this helpful suggestion. As the primary focus of this study is on proposing a new methodological index, including additional regions/countries, is beyond the scope of this article. However, we agree that applying the index to other regions (such as sub-Saharan Africa) could further demonstrate its utility. We have added a note in the discussion section to suggest this as a direction for future research (Section: Discussion and Conclusion, line # 531-536). The proposed index is designed to be broadly applicable to census and survey data across diverse settings.

*Sensitivity to Age Range: While the focus is rightly on 23–62, a brief note on the applicability (or adjustment needs) for other age ranges could broaden utility.*

We thank the reviewer for this thoughtful comment. We already mentioned in the original manuscript (Section: Background, lines # 60-61), and now in the revised manuscript (Section: Introduction, lines # 73-75) that ‘**Younger ages (0–22) and older ages (63 and above) are excluded as the linearity assumption is not plausible for these ages**. However, we have also added a paragraph in the revised manuscript (Section: Methods, lines #224-231) to explain our choice of the 23–62 age range, which is commonly used in demographic studies to minimize the effects of age misreporting among children and the elderly. We also note that the proposed index can be adapted to other adult age ranges depending on data quality and the study context. However, age heaping is typically most pronounced within the selected range, making it the most appropriate for demonstrating the index’s effectiveness.

*Terminology Consistency: Throughout the article, ensure consistent use of symbols (e.g., WI, Wi, RWtot) to avoid reader confusion, especially in equations and figures.*

Thank you for noting this. We have carefully reviewed the entire manuscript to ensure consistent use of abbreviations and symbols. **RWtot** is now referred to as **RMWI** (Robust Modified Whipple Index) throughout the revised text. The original Whipple Index is abbreviated as WI; Noumbissi’s digit-specific indices are denoted as Wi, Spoorenberg’s index is denoted as Wtot, Rogers’ Whipple digit-specific indices are denoted as WIi.

*Minor Comments: Ensure that all figures and tables are referenced in the text and are clearly labeled in the final version.*

We have reviewed the manuscript and ensured that all figures and tables are correctly numbered, labeled, and referenced in the body of the text.

**Response to Reviewer # 2**

We sincerely thank the reviewer for the recognition of the manuscript’s contributions and appreciate the constructive feedback provided to enhance its clarity, impact, and utility. Below, we respond point by point to each suggestion.

*Suggestions for Improvement*

*The manuscript is strong, and the following suggestions are offered in a constructive spirit to enhance its clarity, impact, and utility for the research community.*

*Refine the Terminology*

*While the name “remodified of total modified Whipple’s Index” (and its abbreviation RWtot) is accurate in describing its lineage, it is somewhat cumbersome and may hinder recall and adoption. To facilitate easier reference and citation, the authors might consider a more concise and memorable name. Suggestions include:*

- *Comprehensive Rogers-Whipple Index (CRWI), to credit the methodological foundation.*
- *Ten-Digit Whipple Index (TDWI), to highlight its key feature.*
- *Robust General Whipple Index (RGWI), to emphasize its stability.*

*A more distinct name could help establish the index as a new standard in literature.*

We thank the reviewer for this thoughtful observation and valuable suggestion. We agree that a concise and memorable name could enhance the visibility and adoption of the proposed index. After careful consideration, we have adopted the term “Robust Modified Whipple Index (RMWI)”. RMWI is a more concise, memorable, and meaningful name that aligns with the manuscript title. This revised terminology emphasises both the methodological robustness of the index and its origin in Whipple’s modification tradition. The abbreviation RMWI has been used consistently throughout the revised manuscript.

*Address the Linearity Assumption Trade-off*

*The paper correctly notes that Noumbissi’s method was developed partly to address the “crude” 10-year linearity assumption of Roger’s index , opting instead for a more granular 5-year assumption. The proposed RWtot, by building on Roger’s framework, reverts to this 10-year assumption. The manuscript would be strengthened by a brief discussion explicitly acknowledging this trade-off. The authors should clarify why accepting the broader 10-year linearity assumption is a worthwhile compromise to achieve a mathematically consistent and robust summary index that covers all ten digits without distortion.*

We thank the reviewer for this insightful observation. We acknowledge that returning to the 10-year linearity assumption represents a methodological trade-off. However, the primary goal of the proposed Robust Modified Whipple Index (RMWI) is to ensure mathematical consistency and equal weightage across all ten terminal digits (0–9). This uniform structure avoids the imbalance and overestimation seen in digit-specific indexes calculated by using Noumbissi’s method due to varying denominators in digits 1, 2, 3, and 4 **(see Introduction subsection: Mathematical inconsistencies in Noumbissi’s Indexes**). By adopting a 10-digit framework, the RMWI provides a more balanced and interpretable summary of age heaping patterns across the full digit spectrum. We believe this trade-off supports our goal of developing a robust and generalizable index for assessing age misreporting. In the revised manuscript, we have added a paragraph in the Discussion and Conclusion section (Paragraph 3, lines # 503-513) to explain the rationale behind this choice.

**“One of the key methodological decisions in constructing the Robust Modified Whipple Index (RMWI) ………………………………….……………. mathematically robust and generalizable summary index suitable for use across diverse populations and data sources.”**

*Elaborate on the Proposed Quality Criteria*

*The paper proposes a new set of quality criteria for the RWtot index in Table 2. This is an excellent and necessary step for practical application. However, the derivation of these specific thresholds (e.g. ,“Moderate” for a value of 1.00-1.99, corresponding to 5-9.99% of people reporting an incorrect age) is not detailed. The manuscript would be significantly enhanced by adding a paragraph explaining the rationale behind these ranges. How were these thresholds calibrated? A clearer justification would increase transparency and boost the confidence of researchers looking to apply these standards in their own work.*

The thresholds presented in Table 2 are derived from interpreting the RMWI value as a deviation from ideal terminal digit index values. A value of 0.00 reflects perfect uniformity in terminal digit reporting, indicating no age heaping and misreporting, while higher values represent increasing levels of digit preference or avoidance. A deviation of less than 1% is considered perfectly accurate. Values between 1% and 4.99% are classified as fairly accurate, while the critical threshold falls between 5% and 9.99%, where clear signs of age misreporting are evident, named as moderate, showing approximate data. These cutoffs draw a conceptual parallel with the conventional thresholds used in statistical hypothesis testing. Just as p-values below 0.01, 0.05, and 0.10 represent varying levels of statistical significance in social science research, the RMWI categories provide meaningful breakpoints to gauge the severity of age misreporting. For example, <1% deviation aligns with a highly accurate dataset (analogous to a highly significant p-value), 5–10% marks the zone where misreporting becomes substantively important (like a significant finding), and deviations above 10% indicate severe inaccuracies in age data, similar to a non-significant or unreliable result in statistical terms. We added a Paragraph in the Method section to explain these thresholds immediately after Table 2 (lines # 258-273).

*Enhance Data Visualization*

*The figures are informative, but the comparison between the competing indices could be made more direct and impactful. I would suggest creating a single, combined chart for a key dataset that plots the digit preference indices side-by-side. For example, using the Turkey 1993 data from Table 4 , a bar chart could directly contrast Noumbissi’s W; with the proposed WI; for each digit. This would provide a powerful , at-a-glance visualization of the overestimation issue in the former method and the more plausible distribution of the latter.*

Thank you. We fully agree with the reviewer’s suggestion. To enhance the clarity and visual impact of the comparison between indices, we have added a combined bar chart using the Turkey datasets (Section: Comparison of digit-specific indexes: Wi vs WI, Fig 2) and the simulated dataset (Section: Fig 5). These bar charts give a direct, side-by-side comparison of Noumbissi’s digit-specific indices (Wi) and Rogers’s Whipple’s digit indices (WIi), clearly illustrating the overestimation issue in the Wi and the balanced distribution in WIi.
